# Cumulative Physiological Affect: Thematic Analysis of Forensic Psychological Examination Data from a Homicide Case

**DOI:** 10.17505/jpor.2026.29459

**Published:** 2026-08-24

**Authors:** Aizhan Kudaibergenova, Farit Safuanov, Ainur Urisbayeva, Daulet Duisenbekov, Elmira Kalymbetova

**Affiliations:** 1 Department of General and Applied Psychology, Al-Farabi Kazakh National University, Almaty, Kazakhstan; 2 Department of Communication Skills, Kazakh National Medical University named after S. Asfendiyarov, Almaty, Kazakhstan; 3 Laboratory of Forensic Psychology, V. Serbsky National Medical Research Centre for Psychiatry and Narcology, Moscow, Russia; 4 Department of Clinical and Forensic Psychology, Moscow State Psychological and Pedagogical University, Moscow, Russia; 5 Department of Civil Law and Civil Procedure, Labor Law, Al-Farabi Kazakh National University, Almaty, Kazakhstan

**Keywords:** criminal legal qualification, forensic psychological-psychiatric examination, irresistible impulse, mitigation of responsibility, physiological affect

## Abstract

This study examines a forensic case of homicide within a hierarchical work relationship to determine whether the perpetrator acted in a state of physiological affect. The analysis is based on forensic medical examination materials and employs a qualitative approach grounded in thematic analysis. The findings revealed four themes: (1) a hierarchical environment characterized by humiliation and coercion; (2) gradually intensifying motivational pressure closely linked to dependency, presumed blackmail, and fear of punishment; (3) cumulative emotional exhaustion resulting from prolonged internal stress; and (4) cognitive narrowing. A forensic psychological examination identified manifestations of cumulative physiological affect, suggesting that a significant emotional disturbance influenced the offender’s actions. This case serves as a practical illustration of such psycho-emotional states, which is pertinent for criminologists, forensic scientists, and representatives of related fields.

## Introduction

The investigation of physiological affect has long been an area of significant interest for specialists in forensic psychiatry, psychology, forensic medical examination, and jurisprudence in post-Soviet countries. The term *physiological affect* (PA) is a concept within criminal law describing physiological-biological and cognitive-emotional changes in an individual resulting from specific kinds of highly stressful interactions with other individuals and the environment. These physiological reactions can influence behavior and its legal consequences, carrying legal significance for criminal assessments. Due to historical differences in the development of criminal law, the concept of PA lacks a unified definition across different countries. However, it broadly corresponds to English-language legal concepts such as “extreme emotional disturbance”, “loss of control,” or “heat of passion.” Even within common law^1^ jurisdictions, however, these terms are often ambiguous. All these concepts describe the emotional and psychological state of a perpetrator, although they typically do not imply insanity, and serve as grounds for mitigating criminal punishment. Consequently, these concepts are frequently crucial for investigative bodies, prosecution, defense, and courts as important qualifying factors.

Under the criminal laws of many countries, being in a state of PA is considered a mitigating factor. However, a prerequisite for considering PA as a mitigating circumstance is proof that the criminal act was a reaction to unlawful or immoral behavior by the victim. Such provocations may be singular or systemic. Establishing the presence of PA typically presents a complex challenge for forensic and medical experts, requiring profound knowledge in psychology and psychiatry, as well as substantial practical experience. An individual’s personal traits, background, socio-economic conditions, and the immediate triggers of affect collectively influence the legal assessment of the offense and, ultimately, the court’s decision (Safuanov, 2015; Safuanov & Makushkin, 2013). As each case involving PA is unique, this field continuously demands new empirical data and analytical approaches, highlighting the relevance of this research.

Contemporary research on various aspects of PA in connection with criminal offenses addresses a wide range of issues. These include the criminal-legal and psychological characterization of affect (Korneev & Abdullina, 2023; Mukhacheva, 2016), the peculiarities of qualifying homicide under affective conditions (Bugaev & Melnikov, 2018; Minnikhanova & Shageeva, 2019), and the legal assessment of cases when one individual has caused bodily harm to another due to affective states (Dadaev, 2017b). Scholars explore affect from the perspectives of criminal law, psychology, psychiatry, and physiology (Lazareva et al., 2020) and critically analyze legislation governing diminished capacity, such as in the United States (Ajmal et al., 2023). The present study examines a case from forensic practice concerning a homicide where the perpetrator and victim were in a hierarchical work relationship. The research objective encompasses both theoretical and descriptive components: (1) to clarify the conceptual and comparative legal contours of “physiological affect” within post-Soviet and common law traditions, and (2) to assess, in a specific case from Kazakhstan, whether the perpetrator was in a state of physiological affect at the time of the crime, based on forensic examination data.

### Theoretical Considerations

#### Terminology and legal-contextual interpretation of physiological affect

The accurate interpretation of complex emotional states within criminal proceedings is a central task in forensic medicine. The concept of physiological affect (PA) occupies a distinct position at the intersection of psychology, psychiatry, and criminal law, although it is not entirely clear how the concept should be interpreted. Science in Kazakhstan, as in most post-Soviet states operating at this disciplinary intersection, continues to adhere to terminology established during the Soviet period. In English, the term “irresistible impulse” is commonly used as the closest analog^2^.

The contemporary interdisciplinary perspective views physiological affect as a more complex phenomenon than a simple short-term emotional reaction or an isolated psychophysiological episode. Instead, it can be conceptualized as an “extreme experience” (Mihalits, 2025). Affective states are understood as dynamic processes that unfold across multiple stages, from stimulus exposure to cognitive perception and interpretation, emotional experience, and finally to behavioral and cognitive outcomes (Elfenbein, 2006). In psychological terms, these stages represent interrelated motivational and cognitive processes that progressively transform a stressful situation into an emotionally charged episode. Cognitive-emotional aspects include the perception of injustice, a threat to social standing, humiliation, fear of punishment, or the inability to resolve an escalating conflict. These factors are likely to amplify internal tensions and heighten the perceived urgency of action. A substantial body of empirical evidence indicates that factors such as frustration and prior trauma correlate with violent conflict (Lolai et al., 2025). Concurrently, cognitive mechanisms are activated: the individual reappraises the situation, focuses on the perceived source of provocation, and gradually narrows the range of available behavioral options. In this regard, the processes leading to homicide may be partially analogous to those preceding suicide, in which both impulsive and cumulative mechanisms have been identified (Bozzay et al., 2025). The underlying mechanisms of this interaction between cognition, emotion and motivation, and their implications for consciousness and decision-making processes, are subject to theoretical analysis in cognitive neuroscience (Tucker et al., 2025). This line of inquiry may help elucidate the processes underlying cumulative physiological affect. Prolonged stress is characterized as a multi-stage process involving alarm, resistance, and exhaustion, accompanied by a range of physiological and psychological changes (Ovsiannikova et al., 2024). However, this does not imply an absolute loss of behavioral control. On the contrary, the structure of behavior is preserved under such conditions, albeit constrained by the predominant role of emotionally charged interpretations and beliefs.

Cumulative physiological affect should be interpreted not as a limited temporal aspect of affect, but as an independent procedural structure of its formation. This structure is typically characterized by a gradual escalation of emotional tension, the development of internal conflict, and a state of psychological exhaustion that concludes with the acute release of affective energy. In many cases, a growing sense of hopelessness or entrapment emerges, further intensifying motivational pressure and constraining cognitive flexibility. Notably, such cumulative processes should be distinguished from temporary disruptions in consciousness, which are characterized by impaired reflective reasoning and a diminished ability to accurately assess motives. These disruptions may be accompanied by impulsive actions and emotional reactions that weaken conscious self-control (Liana, 2024).

In common law countries, “irresistible impulse” typically describes a temporary loss of legal sanity or capacity. In contrast, within the legal and psychiatric systems of Kazakhstan and several other post-Soviet countries, a loss of sanity is not a criterion for applying the definition of “physiological affect” (PA). Therefore, the role of PA as a mitigating circumstance in sentencing has received relatively little consideration in the legal scholarship of common law countries in recent decades. This may be attributed to the system of judicial precedents, which introduces greater variability into forming a unified understanding of PA compared to civil law countries. Jury trials and broader judicial discretion further contribute to this complexity (Felthous, 2010). In some U.S. states, irresistible impulse is not recognized as a mitigating factor (California Legislative Information, 1982). Nevertheless, U.S. judicial practice generally permits the consideration of irresistible impulse during investigations and sentencing. For instance, in the 1993 case of Lorena and John Bobbitt, Lorena Bobbitt inflicted serious bodily harm on her husband due to an “irresistible impulse” – a genesis bearing similarities to the cumulative formation of PA. The jury played a pivotal role in the proceedings, resulting in her acquittal (McDonough et al., 2023).

#### Irresistible impulse, insanity, and judicial discretion

The debates surrounding the interpretation of *irresistible impulse* (IR) within English-language scholarship have a long history. The historiography of the insanity defense traditionally presents a contrast between lawyers and doctors and between law and medicine. The foundational precedent for applying the category of irresistible impulse is rooted in the M’Naghten Rules of 1843, which intended to determine whether an individual was mentally ill at the time of an offense and thus not punishable for it. However, since their adoption, the Rules faced criticism from medical professionals, who argued that an individual may know an act is wrong and yet be unable to control their actions (Finnane, 2012).

In English-language literature, irresistible impulse is frequently treated as an attribute of insanity. However, highprofile cases such as that of L. and J. Bobbitt present interpretive challenges for jurists outside common-law systems. The degree of insanity ascribed to Lorena Bobbitt, who was accused and subsequently acquitted, remains questionable. Considering subsequent events – her active social life and the absence of relapses, long-term hospitalization, and isolation – irresistible impulse cannot be perceived solely as a symptom of insanity (and, by consequence, of incapacity or limited capacity). Under U.S. legislative frameworks, judicial discretion plays a decisive role, and irresistible impulse can be considered outside the context of a mental disorder (insanity).

#### Comparative affective-legal constructs in common law systems

Beyond the descriptive context, forensic and related sciences in common law countries distinguish several additional forms that are conceptually proximate to PA. These include *extreme emotional disturbance* (more typical for the United States) (Johnston et al., 2022; Tanaka, 2022), *loss of control* (primarily in England and Wales) (Legislation, 2009), or *heat of passion* (United States) (Johnston & Leahey, 2022; WEX Legal Information Instituted Dictionary, 2022). Despite differences in doctrinal approaches, all these constructs reflect a unified procedural logic. Affect is interpreted not as an instantaneous emotional reaction but as a state arising from a complex interplay of prolonged emotional tension, motivational conflicts, cognitive appraisal, and external provocation. This interplay ultimately leads to a breakdown in self-regulatory mechanisms.

The term “extreme emotional disturbance” is recognized as a partial defense (a mitigating circumstance in the terminology of the Soviet/post-Soviet criminal law school) in murder cases within some common law jurisdictions (e.g., under the U.S. Model Penal Code §210.3). This defense typically requires proof that the accused was under the influence of strong emotions and lost self-control at the time of the offense (Johnston et al., 2022). The term “loss of control” generally denotes an inability to control one’s impulses and behavior due to strong emotions – a condition in which an individual cannot inhibit their reactions (Clark & Kruse, 1990). This concept functions as a partial defense in criminal law in England and Wales, codified by the Coroners and Justice Act 2009, §§ 54–56. It replaced the prior doctrine of “provocation” and applies to murder convictions (Bergelson, 2021). Notably, this category does not require the presence of a mental illness.

The term “heat of passion” is another common law concept closely related to PA. In jurisprudence, it is considered a mitigating factor for a defendant who claims to have acted under uncontrollable rage, terror, or fury during the alleged crime, particularly if provoked by the victim. The heat of passion defense serves to negate the malice element required for a murder prosecution. In *United States vs. Visaniz,* it was held that the prosecution must prove the absence of heat of passion beyond a reasonable doubt to establish malice (WEX Legal Information Instituted Dictionary, 2022). All three constructs are comparable to PA in several key respects. Nonetheless, their application in case law accentuates judicial discretion, expanding the authority of judges and juries to determine whether the relevant psychophysiological state was present during a crime (Table 1).

**Table 1:** Comparison of “Physiological Affect” and Analogous Legal Concepts

Concept	Sanity Status	Pathological Status	Mitigating circumstance
***Physiological affect***	Yes	No	Yes
***Irresistible impulse***	Varies by jurisdiction	Varies by jurisdiction	Yes (may support an insanity defense)
***Extreme emotional disturbance***	Yes	No/ Partially	Yes
***Loss of control***	Yes	No	Yes (requalification for manslaughter)
***Heat of passion***	Yes	No	Yes (requalification for manslaughter)

#### Post-Soviet doctrinal and psychological conceptualizations of affect

Within specialized Russian literature, the most comprehensive definition of PA, reflecting its conceptualization in Kazakhstani law and medicine, is provided by the Great Russian Encyclopedia:.

Physiological affect is a type of forensic-psychological expert concept of ’affect.’ It is a swift and turbulent emotional reaction of an explosive nature that arises suddenly in the accused in response to a psychologically traumatizing impact from the victim. This reaction is accompanied by a sharp impairment of their capacity for the conscious and volitional regulation of their criminal actions. An expert conclusion that the accused was in a state of physiological affect at the time of the alleged act can be used by the court to qualify a sudden and strong emotional disturbance (affect). Such a disturbance, caused by violence, abuse, severe insult, or other unlawful or amoral actions of the victim, may constitute an element of the offense. (Kravets, 2022, authors’ translation).

A similar definition appears in the commentary to Article 98 of the Criminal Code of the Republic of Kazakhstan (“Murder Committed in a State of Affect”):

Affect is a special mental state of a person characterized by its brief and rapid onset, strong and profound emotional experience, vivid external manifestation, narrowing of consciousness, and reduced control over one’s actions. Strong emotional disturbance is not considered a psychiatric disorder and is not regarded as a medical criterion for insanity. Therefore, it is sometimes referred to as physiological affect, in contrast to pathological affect, where intense emotional experience leads to a complete loss of consciousness, thus excluding sanity. Physiological affect does not deprive a person of the ability to be aware of their actions but significantly impairs self-control and critical assessment of decisions. (Borchashvili, 2007, authors’ translation).

A similar approach to that of Kazakhstan in defining PA and affective states is used in Ukrainian law and science. Notably, at the regulatory level, the concept of “affect” is not employed; it is replaced by the term “strong emotional disturbance” (Gutorova & Baylov, 2017; Veresha, 2017). In contrast, legal doctrine, forensic medicine, psychiatry, and related scholarly fields do not avoid using “affect,” “physiological affect,” and corresponding terminology.

In the psychological scholarship of post-Soviet countries, discussions on affective states often refer to the foundational work of the prominent Soviet psychologist S. Rubinstein. He defines affect as a rapid and explosive emotional process that can precipitate actions beyond conscious voluntary control, culminating in a discharge (Rubinstein, 2005). According to another key Soviet theorist, I. Kudryavtsev, a state of affect is characterized by intense and relatively brief emotional experiences, accompanied by pronounced motor and visceral manifestations (Kudryavtsev, 1988). The difference between these interpretations lies primarily in the degree of intensity ascribed to affect. The former emphasizes characteristic features indicative of a disruption in conscious voluntary control of actions, whereas the latter lacks such specificity. Collectively, these definitions encompass the full spectrum of affective reactions, including classical and cumulative variants (Savko, 2019). Ukrainian legal psychology distinguishes between classical and cumulative forms of physiological affect. Their sole distinction lies in the duration of the initial phase. In the classical type, the initial phase is minimal in duration, and the discharge occurs almost instantaneously. This classical form is not preceded by prolonged conflicts or psycho-traumatic situations. Both types exhibit identical manifestations during the discharge phase: an emotionally colored narrowing of consciousness and self-control, intense and disruptive behavioral reactions, and similar symptoms. For both, the hallmark during the discharge phase is its sudden onset, observable to the subject and external witnesses. Under Ukrainian law, either the cumulative or the classical form of physiological affect can serve as a valid basis for mitigating punishment (Savko, 2019).

## Method

### Case Materials

The case study method was employed as a methodological framework to examine a specific case involving the commission of a serious crime by an individual purportedly experiencing physiological affect during the offense. As the case occurred in Kazakhstan, the contextual focus pertains to the legislation and forensic psychological practice of this jurisdiction. The uniqueness of the case precludes direct comparison for analysis; however, foreign expertise is referenced to analyze certain phenomena. Under Kazakhstani criminal law, physiological affect is recognized as a mitigating circumstance that does not exclude criminal liability. It is defined as a state of strong emotional agitation arising in response to an unlawful act, in which the individual largely retains the capacity to understand and control their actions.

The documentary basis of the research consists of primary sources, specifically the Expert Conclusion issued by the Institute of Forensic Expertise^3^ following a comprehensive stationary forensic psychological-psychiatric examination (hereinafter referred to as the Expert Conclusion). An expert conclusion is a standardized instrument in Kazakhstan’s expert practice, regulated by the country’s relevant legislation and issued by competent authorities in accordance with legal procedures. The cited examination was conducted in compliance with forensic research methods approved by Order No. 331 of the Minister of Justice of the Republic of Kazakhstan, dated March 30, 2017.

The Expert Conclusion is grounded in established methodological and reference sources, including the ICD-10 (International Classification of Diseases, 10th Revision; World Health Organization, 2004). The examination employed clinical-psychopathological research methods, which comprised anamnesis, follow-up, medical observation, clinical interviews, mental state description, and analyses of symptoms of potential mental disorders. This was combined with neuropsychological and psychodiagnostic assessment methods. The assessment of mnemonic functions utilized tests such as “Learning 10 Words,” the “Pictogram” method, and the Benton Visual Retention Test. The evaluation of cognitive activity involved tasks of exclusion of objects, comparison of concepts, explanation of proverbs, sequencing of plot pictures, etc. To maintain clarity in the argument’s progression, the descriptive section of this manuscript follows the sequence presented in the original source (the Expert Conclusion).

### Thematic Analysis

The empirical part of this research involved a thematic analysis of forensic psychiatric examination documents. This analysis aimed to identify recurring and legally or clinically significant semantic elements from the following sources: (1) expert conclusions, covering both outpatient and inpatient forensic psychiatric examinations, along with data from the neuropsychological and psychodiagnostic assessment; (2) verbatim witness testimonies; and (3) materials procured during investigative procedures. Thematic analysis was conducted in accordance with the guidelines of Braun and Clarke (2006) and involved the following stages: familiarizing with data, generating initial codes, searching for themes, reviewing the themes, defining and naming the themes, and producing the report.

In the initial phase of thematic analysis, the entire corpus of documents was carefully examined through repeated readings to fully capture the breadth and depth of the data. The corpus included interview transcripts, materials from the internal investigation; a description of the case circumstances and biographical information on the defendant; and the conclusions of outpatient and inpatient comprehensive inpatient forensic psychological-psychiatric examinations, including data from a neuropsychological and psychodiagnostic assessment. During this stage, preliminary analytic notes were compiled to document emerging ideas and potential patterns relevant to the research question. This approach enabled immersion in the material and the preliminary identification of key semantic patterns. Particular attention was paid to the following aspects: (1) the relationship between the defendant and the victim; (2) the organizational and psychological atmosphere within the unit; (3) the emotional and behavioral characteristics of the defendant before, during, and after the incident; and (4) expert interpretations of his mental state. As part of the analytical process, a written summary of the expert opinions was compiled using data from the forensic psychiatric examination. Although it is not possible to include the full summary in the manuscript due to existing ethical and legal constraints, it can be made available upon reasonable request.

#### Generating initial codes

Subsequently, the data were systematically coded using an inductive, semantic, and interpretive approach. The analysis encompassed both explicit semantic units contained within the documents and the deeper psychological aspects derived from witness testimonies, internal investigation findings, and expert evaluations. Coding was performed iteratively across the entire dataset, with key text segments (data excerpts) highlighted and assigned preliminary codes.

Data excerpts from documents and witness testimonies were distributed across each code to organize the material into analytically meaningful groups. A sequential examination of patterns across documents and testimonies revealed a high degree of substantive consistency in the codes. Coding and thematic analysis were conducted jointly by the authors. In the initial phase, coding was performed independently by each author, and any discrepancies were resolved through discussion. The final themes were determined by consensus to ensure the reliability and consistency of the analysis.

#### Searching for themes

In the third stage of thematic analysis, codes with similar meanings were grouped into initial thematic clusters to move from discrete semantic units toward more generalized analytical patterns. At this stage, the coded data segments corresponding to each code were collated and analyzed collectively to determine how these codes might coalesce into broader thematic structures. This process yielded the following preliminary themes: (1) a toxic hierarchical environment and systematic humiliation; (2) moral pressure and involvement in problematic work-related relationships; (3) accumulation of emotional tension; (4) a sense of hopelessness and narrowing of situational awareness; (5) cognitive narrowing and acute affective discharge; and (6) expert assessment of the condition as cumulative physiological affect. These themes were established as working analytical categories subject to further verification for comprehensiveness, logical coherence, and distinctness.

#### Reviewing the themes

In the fourth stage, the preliminary themes were examined against the entire corpus of data pertaining to the case. First, the coded data extracts associated with each theme were reexamined to ensure internal coherence and analytical integrity. The analysis focused on evaluating the empirical support for each theme, removing redundant content, and identifying the distinct role of each theme in elucidating the investigated process. Second, the entire dataset was re-analyzed to confirm that the thematic structure accurately reflected the patterns present in the data. The results indicated that several initial themes were closely interrelated, representing different facets of a single overarching process. For instance, themes pertaining to moral pressure, feelings of hopelessness, and the buildup of internal tension were reconceptualized as sequential components of a unified process – cumulative physiological affect. The analysis further established that the organizational context, interpersonal humiliation, and expert conclusions do not function as discrete parallel elements. Rather, they constitute complementary levels of analysis: the external social context, the internal psychological dynamics, and the professional interpretation provided by forensic experts. This refinement simplified and conceptually clarified the analytical structure. The validity of four key themes, which most accurately reflect the totality of the collected data, was confirmed.

#### Defining and naming the themes

At this stage, the analysis focused on identifying the essence of each identified theme and clarifying the specific aspects of the phenomena they represent. The relationships between themes were also examined to ensure they formed a coherent explanatory model of the psychological process underlying the case.

### Anonymization and Case Designations

The coding scheme developed in this study classifies the participants in the proceedings, including the main actors, witnesses, and the victim. The system uses the designation *INDIVIDUAL* (abbreviated as “*I*”) to identify persons directly connected to the case: *I1* represents the accused, i.e., the individual alleged to have committed the offense, while *I2* denotes the victim, i.e., the person who suffered harm as a result of the accused’s actions. Witnesses are identified using the designation *WITNESS* (*W*), with numerical subscripts (e.g., *W1* to *W4*) serving to distinguish individuals in this category.

### Ethical Considerations

This study presents a forensic analysis of cumulative physiological affect based on a specific case subjected to forensic medical examination. The study adheres to the ethical standards for scientific research outlined in documents such as the Belmont Report (1979), the Declaration of Helsinki (1964), the European Code of Conduct for Research Integrity (Revised edition 2023), and other internationally recognized principles. Specifically, the following measures were implemented:

a) Full anonymization: All personal data, including names, dates, locations of events, and other identifying features, were excluded or replaced with conventional designations. This anonymization was performed in accordance with established principles for protecting confidential information in scientific practice.
b) Non-intervention: The study was purely analytical and involved no interference with the privacy or the mental or physical state of any individuals. The analysis was conducted using documentation from a concluded legal case.
c) Consent: As the analysis concerns a case finalized through formal judicial proceedings, with all information sourced from archived forensic materials, obtaining individual informed consent was not feasible. Every precaution has been taken to prevent the identification of individuals.

Given the research topic and methodological approach, this study did not require additional ethical approval from authorized institutions. It did not involve interference with privacy, experiments on human subjects, or the use of biological samples for clinical or scientific purposes. The research is fully consistent with the Code of Ethics of the Forensic Expert of the Republic of Kazakhstan, the Model Ethical Code for the Psychologists of the Republic of Kazakhstan, and other relevant documents (Minister of Justice of the Republic of Kazakhstan, 2017; Psychology Selena, 2022).

The study incorporates quotations and excerpts from the Expert Conclusion. These are preserved without alteration (with editorial notes in specific cases) to accurately provide information from pre-trial investigation materials, which contain subjective nuances essential for contextualizing the events. Personal names and institutional identifiers have been replaced with generic designations such as “INDIVIDUAL” (abbreviated as “I”), “WITNESS” (“W”), and “VICTIM” (“V”). Geographical locations are represented by Latin letters.

## Results

**Figure 1:**
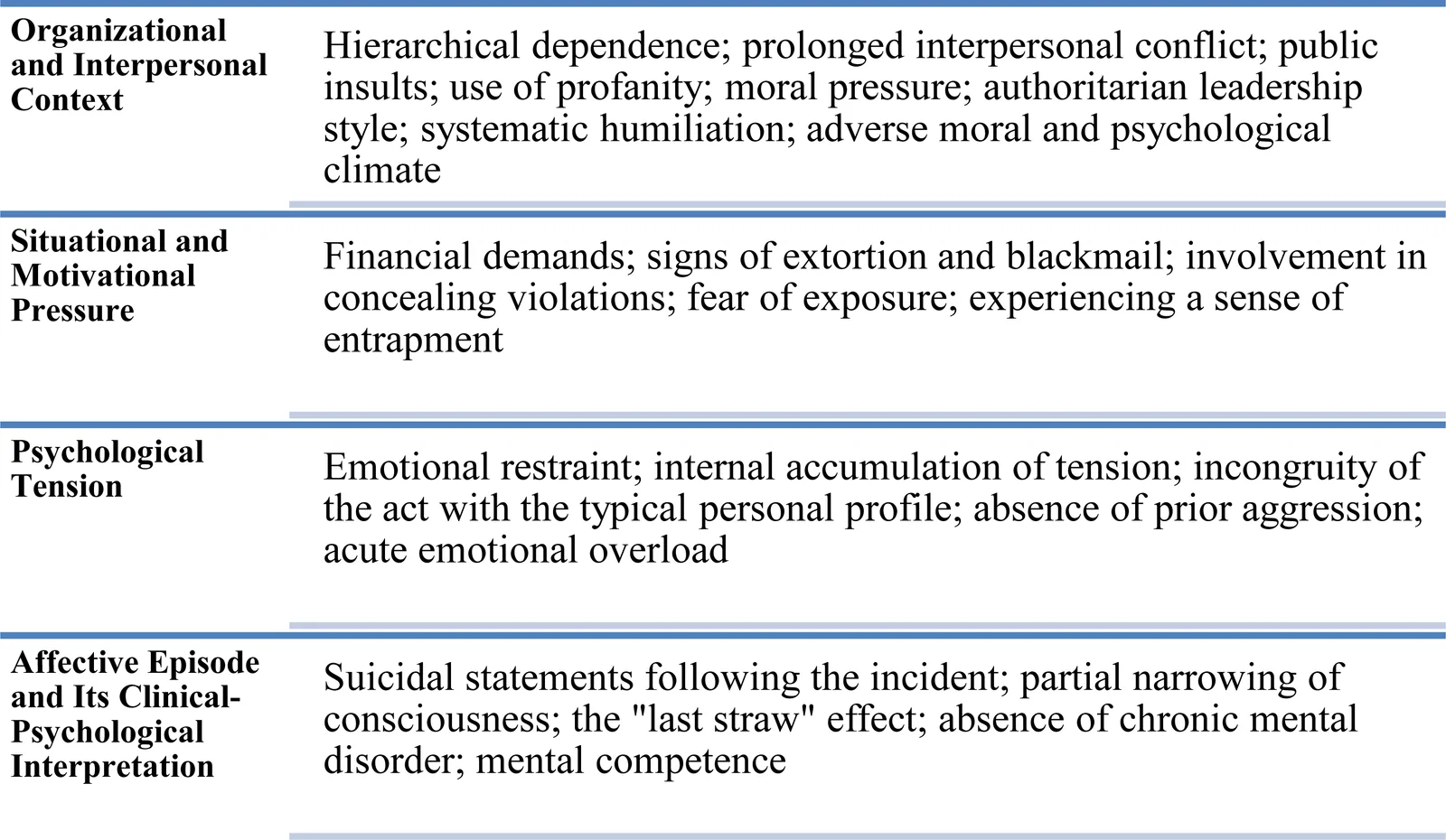
Codes as identified and categorized during systematic coding The thematic analysis resulted in four key themes (see Table 2).

The coding identified recurring categories, which were summarized into four groups, as presented in Figure 1.

**Table 2:** Final themes resulting from thematic analysis of the case materials

Theme	Content	Supporting Data Excerpts	Analytical Interpretation
1. Hierarchical environment characterized by humiliation and coercion	The complex and strained interpersonal and professional context in which the defendant remained subordinate to the victim over an extended period. This theme reflects manifestations of rudeness, public humiliation, sustained moral pressure, violations of hierarchical norms, and an authoritarian management style.	*W2* stated that *I2* “occasionally used profane language and obscenities toward *I1* in the presence of subordinate officers, apparently aiming to morally degrade him.” *W3* reported that *I2* “was somewhat arrogant… quick-tempered” and frequently criticized I1 unjustifiably. *W4* characterized *I2* as “arrogant, insolent, greedy… rude, prone to using foul language toward his subordinates, often insulting and humiliating them publicly.” The investigation materials noted “an unhealthy moral and psychological climate within the MDTU’s military unit.”	The events did not take place in a neutral environment but rather within a persistently traumatizing workplace context. The decisive factor was not an isolated conflict but a recurring pattern of humiliation and dominance. This environment may have contributed to prolonged emotional tension and diminished the defendant’s capacity to cope with stress.
2. Intensifying motivational pressure	Materials indicate that the defendant found himself under mounting internal tension related to dependency on the victim, alleged extortion, involvement in concealing violations, and fear of potential consequences. This theme encompasses feelings of hopelessness, injustice, and fear of being accused.	*W2* relayed the statements of *I1* that *I2* “had repeatedly burdened him with financial demands [interpreted as extortion or blackmail].” *I1* also stated that his actions were motivated by *I2’s* intention to accuse him of embezzling “3,000,000 tenge.” *W4* described situations in which *I2* “repeatedly coerced him into unlawful actions against his will, threatening dismissal and employing intimidation,” and *I1* was aware of these episodes and signed documents without verification. The report notes: “Clearly, this situation, coupled with the realization that continuation of military service outside the local military management system was not possible for him, compelled [*I1*] to seek a way out of the situation and prompted him to make the corresponding decision.”	The motivational dimension of the affective act is evident. The documents suggest that the defendant perceived the situation as more than a mere conflict with a superior. An escalating sense of threat, the inability to distance himself from events, and fear of being accused resulted in a growing feeling of hopelessness (potentially a key factor preceding the affective discharge).
3. Cumulative emotional exhaustion	This theme describes how the defendant psychologically coped with prolonged pressure. According to the materials, he was not predisposed to open aggression; rather, he was described as calm and balanced, tending to suppress emotions. This pattern may have facilitated the gradual accumulation of internal tension.	*W2* described *I1* as “diligent, honest, and conscientious,” “calm and balanced.” *W3* characterized him as “calm, cheerful, sociable” and emphasized that he “had never observed any kind of negativity or aggression from him.” However, *W3* noted that *I1* did not disclose all conflict episodes, “keeping everything to himself.” In a telephone conversation after the incident, *I1* told *W2* that he “could no longer endure his life difficulties.” According to *W2, I1* “expressed an intention to shoot [himself] and had already removed his socks to use his toe to press the trigger of the rifle.” A neuropsychological and psychodiagnostic assessment revealed “emotional tension, stress, and frustration” and documented “the accumulation of emotional tension in the subject.”	This theme distinguishes cumulative affect from impulsive aggression as a personality trait. The materials consistently portray an individual not inclined toward cruelty or open conflict but prone to accumulating internal tension. Rather than manifesting as sudden outbursts of irritation, this psychological dynamic operates through the sustained internalization of stress, which ultimately depletes selfcontrol resources.
4. Cognitive constriction and affective discharge	This theme examines the transition from accumulated tension to an acute episode. Evidence suggests partial narrowing of consciousness, fixation on the traumatic stimulus, and diminished capacity for rational control of behavior at the moment of the offense.	A neuropsychological and psychodiagnostic assessment indicated: “Chronic emotional tension facilitated an affective outburst analogous to the ’last straw’ phenomenon.” It further noted that the victim’s behavior evoked “acute feelings of fear, offense, and insult,” accompanied by “a partial narrowing of consciousness.” The inpatient forensic commission concluded that at the time of the act, *I1* experienced “pronounced emotional tension of cumulative genesis,” which “significantly influenced his consciousness and behavior and reached the level of a physiological affect.” The final analytical summary observed that *I1* “could not rationalize his motives at the moment of the crime and thereafter.” Additionally, after committing the offense, *I1* stated that he had “six bullets, one for myself,” reflecting articulated thoughts of suicide.	Accumulated emotional tension heightened feelings of threat and humiliation, affecting cognitive processing: attention and situational appraisal narrowed, the ability to perceive alternative solutions diminished, and behavior became governed by immediate affective response. This theme establishes the connection between the objective case data and the expert conclusion regarding cumulative physiological affect. Additional evidence supporting the presence of an affective state is provided by the accused’s voiced suicidal intentions. This may indicate that the incident was part of a profound crisis state characterized by emotional overload, a sense of hopelessness, and cognitive constriction. Importantly, the subsequent change in behavior, including interactions with relatives and voluntary surrender to authorities, may reflect a gradual restoration of cognitive control following the most intense phase of the affective episode.

### Summary of the Analysis

The thematic analysis revealed that the incident was not an instantaneous outburst of aggression but rather the result of a prolonged psychotraumatic situation. Over an extended period, the defendant had been immersed in an environment characterized by a power imbalance, stemming from an authoritarian management style and sustained moral pressure from the victim. Progressively intensifying factors of motivational pressure were also identified, attributable to the defendant’s involvement in conflict-laden and potentially unlawful workplace activities. According to case materials, these factors included financial demands, threats of accusations regarding property embezzlement, and coercion to participate in concealing violations. Such circumstances may have exacerbated the internal crisis, fostering a sense of injustice and entrapment in the defendant. In his perception, no escape from the situation was possible without consequences. The interpersonal conflict gradually evolved into a professional and moral trap, compounded by a pervasive feeling of hopelessness.

The analysis further suggested that significant psychodynamic processes may have contributed to the accumulation of emotional tension. Witnesses and experts consistently described the defendant as a calm and balanced individual not predisposed to aggression. However, his tendency to avoid discussing personal difficulties, encapsulated by one witness’s observation that he “kept everything to himself,” appears to have facilitated the buildup of internal stress. Psychological evaluations corroborated indications of prolonged emotional strain, frustration, and sustained psychoemotional pressure. Forensic psychiatric materials documented a state of pronounced emotional tension arising from cumulative stress. At the critical moment, the defendant experienced a partial narrowing of consciousness and a sharp affective response. This acute state was also manifested in the suicidal intentions expressed by the accused immediately after the commission of the offense, including statements emphasizing a readiness to commit suicide. These circumstances may indirectly suggest that the affective episode was part of a deeper crisis state, accompanied by feelings of hopelessness and emotional collapse. He suffered from no chronic mental disorder and retained the capacity to comprehend his actions; nevertheless, his emotional state substantially influenced his behavior at the time of the incident.

## Discussion

The thematic analysis identified four patterns preceding the commission of the crime. These included a hierarchical environment marked by humiliation and coercion, intensifying motivational pressure, cumulative emotional exhaustion, and cognitive constriction culminating in affective discharge. The findings suggest that the crime in question cannot be regarded as an isolated outburst of aggression. Rather, it represents the outcome of prolonged psychotraumatic exposure resulting from the mutual reinforcement of organizational, interpersonal, motivational, and cognitive-affective factors. This aligns well with the concept of physiological affect, as defined above.

N. Rakhmetullina, Chair of the Judicial Collegium for Criminal Cases of the Supreme Court of the Republic of Kazakhstan, notes that defendants and their defense counsel often invoke the state of affect in complaints and motions, yet establishing its presence in practice remains challenging. Determining whether and for how long an accused was in a state of affect necessitates a forensic psychological and psychiatric examination (Rakhmetullina, 2024). The Supreme Court’s regulatory resolution “On Forensic Examination in Criminal Cases” stipulates that an expert’s report must be examined and assessed by the court (Online Zakon, 2004). When reviewing such a report, it must be recognized that it holds no inherent advantage over other evidence nor possesses preclusive authority; it is subject to analysis, comparison, and evaluation in conjunction with all case materials. Thus, while the court is obligated to assess the expert report and retains the right to disagree with its conclusions, it must, in such instances, order a supplementary or repeat examination. Lacking specialized scientific knowledge, the court cannot establish the presence or absence of an affective state without a corresponding expert conclusion. The judge further notes practical cases where multiple expert examinations have concurrently testified that an individual was in a state of affect. In such cases, courts must meticulously examine not only the operative section but also the research portion of the conclusion and consult relevant scientific primary sources. It is obligatory to interrogate the experts with detailed questions covering all stages of the affective state: the pre-affective stage, characterized by increasing emotional tension; the affective “explosion” or emotional outburst; and the post-affective stage (exhaustion, depression, feeling of remorse) (Rakhmetullina, 2024).

The first theme identified in the thematic analysis (a hierarchical environment characterized by humiliation and coercion) reflects the long-term organizational and interpersonal context in which the defendant was situated. Witness testimonies confirm recurrent instances of public insults, the use of profane language, and an authoritarian communication style. Internal investigation materials document an adverse moral and psychological climate. Such a situation illustrates the context where cumulative affect can arise, not from a single incident but from a prolonged psychotraumatic situation with escalating emotional tension (Rakhmetullina, 2024).

The second theme suggests that the interpersonal conflict assumed the form of a trap, which in turn may have escalated emotional tension. Witness testimonies and expert evaluations describe the defendant as a calm and conscientious individual, not predisposed to aggression or conflict. One witness noted that the defendant preferred not to discuss his difficulties; such an approach to emotional management may have led to the accumulation of stress. Thus, the observed pattern was not one of chronic aggressiveness but rather a gradual buildup of internal tension leading to cumulative affect. The incident could be interpreted as an affective reaction accompanied by intense emotions of fear, offense, and humiliation, as well as partial narrowing of consciousness, in the context of a conflict unfolding within conditions of unequal power relations in the workplace. The dynamics of escalation in such contexts appear as follows: prolonged external pressure within a hierarchical structure engenders feelings of hopelessness and intensifies internal emotional tension. Over time, this may lead to cognitive constriction and culminate in emotional discharge.

Within modern integrated criminology – particularly in the American school represented by Gottfredson and Hirschi (2022) – criminal behavior is explained through the lens of ineffective individual self-regulation. This concept is examined in detail by American psychologists Baumeister, Vos, and Schmeichel (Baumeister & Vohs, 2007; Vohs & Baumeister, 2004; Vohs & Schmeichel, 2003). Their analyses shift the explanatory focus for criminal behavior from external social factors to internal psychological mechanisms of behavioral control, primarily self-regulation. Self-control is defined as an individual’s ability to restrain impulses, delay gratification, anticipate consequences, and align actions with social norms. A criminological explanation for ineffective self-regulation suggests that criminal behavior arises from an individual’s failure to manage impulses and desires in specific situations, even when aware of potential adverse outcomes. This links criminological theories with psychological research on willpower, self-control, and decisionmaking, framing crime as a specific instance of a broader dysfunctional behavioral regulation.

Gottfredson and Hirschi’s (2022) theory has been widely criticized for its excessive psychologism and its narrow focus on individual characteristics at the expense of social structures. Critics also note its disregard for power dynamics and institutional violence, as well as its reduction of social conflict to individual traits (Burt, 2020; Enzmann, 2022). As a result, this theory does not hold a dominant position in European criminology. Although the General Theory of Crime occupies a prominent place in Anglo-American scholarship, it is perceived more cautiously and selectively within postSoviet criminological research (Vasiliev, 2019). Post-Soviet practice places greater emphasis on situational dynamics, affective states, and forensic reconstruction of the offender’s mental state during the crime. Unlike the American criminological tradition, which prioritizes stable dispositional traits such as self-control, this approach facilitates the identification of procedural and situational mechanisms underlying behavioral escalation. This distinction is not evaluative and does not preclude comparability with integrated theories of self-regulation. On the contrary, it highlights the potential for their empirical refinement within different legal and methodological contexts.

In an increasingly globalized world, criminal cases with cross-border dimensions are becoming more frequent. Therefore, understanding how physiological affect is interpreted in different legal systems is crucial for international cooperation, extradition issues, and the harmonization of criminal law. Future research may focus on comparing the legal and scientific formalization of affective states across jurisdictions and their forensic evaluation in selected countries.

## Data Availability

As part of the analytical process, a written summary of the expert opinions was compiled using data from the forensic psychiatric examination. Although it is not possible to include the full summary in the manuscript due to existing ethical and legal constraints, it can be made available upon reasonable request.

## Footnotes

1 Common law is a legal system that develops primarily through binding judicial decisions and precedents, rather than statutes or codified laws (as in civil law). The presiding judge determines which precedents to apply in deciding each new case.

2 Black’s Law Dictionary (United States) defines “irresistible impulse” as follows: “Primarily used in criminal law, this term denotes an impulse to commit an unlawful or criminal act that the patient cannot resist or overcome, because insanity or mental illness has destroyed his freedom of will, his ability for self-control and choice as to his actions.” (Note: Black’s Law Dictionary, or “Blacks,” is traditionally considered an authoritative legal dictionary in the field of U.S. law) (The Law Dictionary, 2024).

3 The Institute of Forensic Expertise is a specialized agency of the Republic of Kazakhstan, subordinate to the Ministry of Justice of the country and authorized to conduct forensic examinations in criminal, civil, and administrative cases by applying special scientific knowledge. The Institute also conducts research on matters related to forensic examination. Its functions include, among others, establishing an individual’s psychological state, performing complex forensic medical and psychological-psychiatric expert studies, and conducting forensic psychological research on physiological affect (Republican State Enterprise “Forensic Expertise Center” of the Ministry of Justice of the Republic of Kazakhstan, 2025)

## List of abbreviations

PA: physiological affect

ICD-10: International Classification of Diseases, 10th Revision

*I1*: The accused person

*I2*: The victim

*W1*: WITNESS 1

*W2*: WITNESS 2

*W3*: WITNESS 3

*W4*: WITNESS 4

## Action editor

Lars-Gunnar Lundh served as action editor for this article.

## Open access

This article is distributed under the terms of the Creative Commons Attribution 4.0 International License (http://creativecommons.org/licenses/by/4.0/), which permits unrestricted use, distribution, and reproduction in any medium, provided you give appropriate credit to the original author(s) and the source, provide a link to the Creative Commons license, and indicate if changes were made.
